# SureTypeSCR: R package for rapid quality control and genotyping of SNP arrays from single cells

**DOI:** 10.12688/f1000research.53287.1

**Published:** 2021-09-21

**Authors:** Ivan Vogel, Lishan Cai, Lea Jerman-Plesec, Eva R. Hoffmann

**Affiliations:** 1 DNRF Center for Chromosome Stability, Department of Cellular and Molecular Medicine, Faculty of Health and Medical Sciences, University of Copenhagen, Copenhagen, Denmark

**Keywords:** single cell genotyping, SNP array, quality control, machine learning, tidyverse, R package

## Abstract

Genotyping of single cells using single nucleotide polymorphism arrays is a cost-effective technology that provides good coverage and precision, but requires whole genome amplification (WGA) due to the low amount of genetic material. Since WGA introduces noise, we recently developed SureTypeSC, an algorithm to minimize genotyping errors. Here, we present SureTypeSCR, an R package that integrates a state-of-the-art algorithm (SureTypeSC) for noise reduction in single cell genotyping and unites all common parts of genotyping workflow in a single tool. SureTypeSCR is built on top of the tidyverse ecosystem, which facilitates common operations over the data and allows users to create and experiment with the genotyping pipeline. Furthermore, the workflow of SureTypeSCR can also be used for standard genotyping of bulk DNA for batch processing in a single pipeline. SureTypeSCR is avaliable from: https://github.com/Meiomap/SureTypeSCR

## Introduction

Single cell genotyping allows genomic discovery when material is limited such as in preimplantation genetic test of embryos for aneuploidy or monogenic disease. Furthermore, analysis of single cells also facilitates the discovery of heterogeneity of *de novo* mutations and copy number aberrations across a population. ^1- 3^ Whereas genotyping using single-nucleotide polymorphism (SNP) array technology benefits from high precision and good coverage of SNPs and is a cost effective way of reconstructing haplotypes when analyzing bulk DNA from a population of cells, single cell genotyping requires whole-genome amplification (WGA) prior to analysis.

WGA is a necessary step in the workflow due to insufficient amount of DNA in single cells (8 pg) for SNP array analysis, which requires 100 ng or above. ^4^ However, WGA introduces two categories of errors: (1) allele drop out (ADO) and (2) allele drop in (ADI). ADO occurs when WGA fails to amplify one of the alleles such as a heterozygous genotype (AB) is mistakenly genotyped as AA or BB. ADO is common and affects up to 30% of typed SNPs. ^5^ ADI is less frequent than ADO and occurs when an AA or BB genotype is erroneously typed as AB. We previously showed that this occurs when the fluorescence signals of both alleles are suboptimal and an artefact of the normalization procedure. ^6^ Multiple tools have been developed for analyzing the noise due to WGA in the sequencing data, whereas there are few experimental approaches for removing noise in SNP array data. They include increasing the genotyping scores based on the standard algorithms developed for bulk DNA ^7^ or use parental support information to exclude erroneous variants. ^8^ We previously developed a machine learning algorithm (SureTypeSC) that is trained on 28 million SNPs from 104 single cells that improves both recall and precision of the single cell data. ^6^

Currently, analysis of SNP arrays is a multi-step process. The principle of SNP array genotyping by Illumina is measuring allelic ratio represented by red and green channel intensities for each allele (generically known as A and B). The intensities are stored in IDAT files and are then normalized using six-degree affine transformation and in GTC format. ^9^ Illumina's GenomeStudio software is the standard tool for analyzing and quality checking of the genotypes and is compatible with both IDAT and GTC. However, including GenomeStudio in a pipeline with large sample batches can be impractical as the data loading process needs to be curated manually. Tools other than GenomeStudio designed for automated data conversion from IDAT to GTC include AutoCall (for Windows) and IAAP Genotyping CLI (for multiple platforms), both developed by Illumina. IDAT is a proprietary binary format and to our knowledge there is only one tool supporting its parsing - an R package illuminaio. ^10^ Automated feature extraction from the GTC file can be done by Illumina's library IlluminaBeadArray that stores the features in numpy array, ^11^ a data structure that allows convenient programmatic processing. There are tools that directly convert the GTC format to commonly used variant calling format (VCF) either issued by Illumina or available in the bioinformatics community ( gtc2vcf).

SureTypeSC, a Python library developed for precise single cell genotyping, requires optimization of certain parameters as well as manual curation of the GTC files in order to extract the genotype features by a 3rd party software (e.g. Illumina GenomeStudio). As this approach is experimental and requires programming knowledge of Python, we encapsulated the functionality of SureTypeSC together with automated feature extraction from the raw GTC data into an R package called SureTypeSCR. SureTypeSCR follows modern data science principles by using packages from tidyverse ^12^ and allows rapid evaluation, visualization and presentation of SNP array data from single cells.

## Methods

### Metadata

The minimal set of input data for loading the Illumina SNP array data consists of a manifest file, cluster file, sample sheet and a set of GTC files, where each GTC file corresponds to one sample analyzed on the SNP array ( Table 1). Both manifest file and cluster file are issued by Illumina per SNP array type. While manifest file describes SNP markers used on the array, cluster file contains information about genotype clusters per SNP marker gathered from population studies and used for scoring in the GenomeStudio software. ^13^

**Table 1.** The minimal set of input data for SNP array genotyping.

Data	Type	Source
Manifest file	Metadata	Illumina
Cluster file	Metadata	Illumina
Sample sheet	Metadata	Defined by the user
GTC files	Data	SNP array

### Implementation

**Figure 1.**
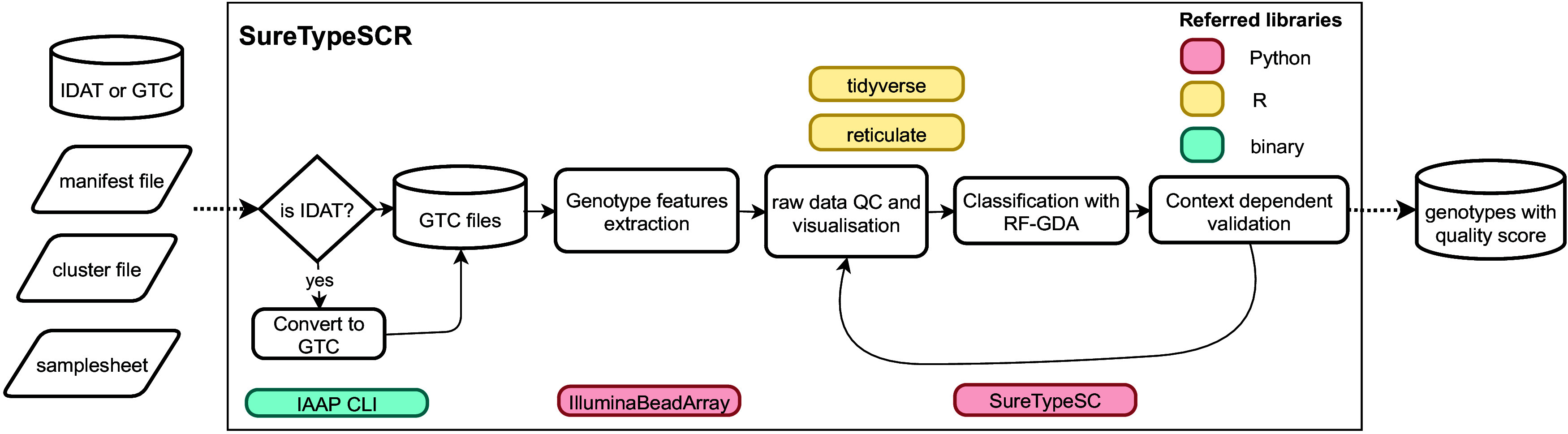
Workflow implemented within the SureTypeSCR package. SureTypeSCR utilizes Illumina's IlluminaBeadArray library to load the metadata ( Table 1) and raw genotype files. In case the data is in IDAT format, SureTypeSCR utilizes Illumina’s IAAP CLI software to convert it into GTC format. SureTypeSCR then implements various functions to check the quality of the data, perform intensity transformation, run dimension reduction algorithm and visualize the results. Subsequently, classification is performed using machine learning algorithm previously trained on large batch of single cell data with known ground truth. ^6^ The algorithm is currently embodied in RF-GDA, which is part of the SureTypeSC Python library. An optional step is context dependent validation that can be implemented within SureTypeSCR in case parental or ploidy information are available.

The core of the package is implemented in a Python library and SureTypeSCR communicates with this library using reticulate. SureTypeSCR further uses Illumina's Python library IlluminaBeadArray to load the GTC files and then utilizes functions from the tidyverse ecosystem (packages dplyr and magrittr) to implement functions for assessing data quality. The data classification process then assigns a quality score to each analyzed single cell genotype ( Figure 1).

### Operation

R (>4.0) and Python (>3.6) are required for installing and running the SureTypeSCR package. The software is installed using devtools. To ensure maximal reproducibility across different platforms, a virtual Python environment is created and all necessary Python dependencies are installed in this environment using reticulate. Subsequently, SureTypeSCR is built, installed and linked to the Python virtual environment The package was tested on three major platforms (Linux/Win/Mac). Data processing times depend on the number of samples in the batch and is estimated at 20s per sample on a single CPU with 4 GB RAM.

## Use cases

To demonstrate functionality of SureTypeSCR, we selected 23 single sperm samples from two families to explore the data and perform genotype classification (GEO database; accession GSE19247). The samples were amplified with multiple displacement amplification and processed on the Illumina Human CytoSNP array. ^8^

### Data initialization and QC

We start the analysis with initializing the package, data and metadata (see Table 1 and code below). The R data package containing the sperm data can be downloaded from GitHub using devtools. Function data(.) then initializes data frame metadata, which stores the family information and other metadata that can be used in the analysis and samplesheet containing path to the downloaded samplesheet with the data. Manifest and cluster file are part of the SuretypeSCR installation. Function scbasic(.) loads the data into an R data frame. We then filter out SNPs, termed intensity only SNPs, that are used to detect copy number variant but do not provide genotyping information (filter(.) and str_detect(.)).


library(devtools)
# install SureTypeSCR from github
devtools::install_github("Meiomap/SureTypeSCR")
# install data package with sperm data (compiled from GSE19247)
devtools::install_github("Meiomap/johnsonspermdata")

library(SureTypeSCR)
library(johnsonspermdata)
# load metadata and samplesheet location
data(metadata,samplesheet)
setwd(system.file("data",package="johnsonspermdata"))

manifest=system.file("files/HumanCytoSNP-12v2_H.bpm",package="SureTypeSCR")
cluster=system.file("files/HumanCytoSNP-12v2_H.egt",package="SureTypeSCR")

df = scbasic(samplesheet=samplesheet, bpm=manifest, egt=cluster)
#filtering out intensity-only SNPs
df %<>% filter ( Chr !=0 & !str_detect (Name ,"cnv"))


**Figure 2.**
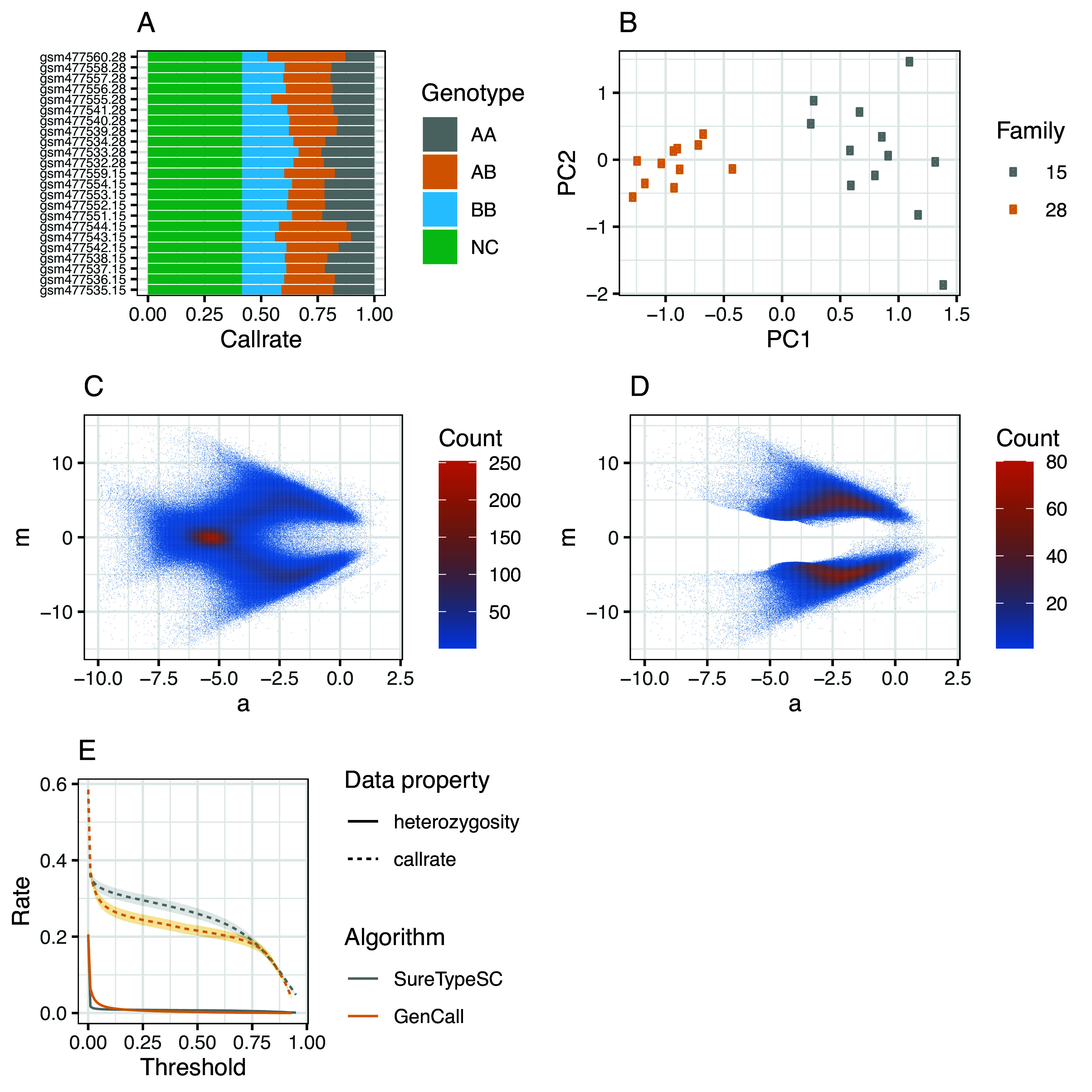
Visual outputs from the analysis of 23 sperm samples with SureTypeSCR. (A) Call rates per individual and genotype calculated as proportions of called genotypes and all genotypes and no calls. No calls represent SNPs with itentisites below a baseline defined in the paper describing the original data. ^8^ (B) PCA analysis accross all SNPs and genotypes from 23 samples that called in every sample. (C) MA plot on normalized intensities across 23 samples, the X axis corresponds to logarithmic average (a) and the Y axis is logarithmic difference (m). (D) MA plot across 23 samples after filtration with SureTypeSC. (E) Effect of used threshold on average heterozygosity (solid line) and average call rate (dashed line) across 23 sperm samples for both, SureTypeSC (grey line) and Illumina GenCall (yellow line). The ribbons represent the standard error of mean.

Calculating call rates per individual and genotype reveals a high degree of heterozygosity ( Figure 2A, AB rates), suggestive of significant ADI, since sperm are haploid cells and there were no aneuploidies reported in these samples: ^8^
df %>%
group_by(individual,gtype) %>%
callrate()


The principal component analysis (PCA) is performed using function plot_pca(.) that returns a ggplot object:
df %>%
plot_pca(features="gtype", metadata=metadata, by_chrom=FALSE)


As shown in the code example above, users can customize which features (columns of the data frame) to use for the PCA analysis with the features parameter. There is an option to customize and add metadata to the ggplot object (currently, family information is supported) and a choice whether the PCA should be run per chromosome (by_chrom parameter) or on the whole data frame. While the per chromosome analysis can reveal aneuploid chromosomes, the latter is useful for validating kinship of the samples. This is demonstrated in Figure 2B, where the 23 sperm samples are separated into two clusters corresponding to two families defined in the metadata.

### Data transformation and classification

Transformation of the intensities into a logarithmic scale minimizes the variability between the SNPs and samples and allows the patterns of the genotyping clusters to be detected. ^6^ In order to evaluate the single cell genotypes using our classification algorithm, we calculate the logarithmic difference and logarithmic average of the intensities (m and a, respectively, Figure 2C). The following code performs the data transformation by adding four additional columns to the original data frame, two for the raw intensities and two for the normalized intensities for the X and Y channels. The user can then control the plotting by adjusting the fraction of points to be visualized, whether a smoothing spline should be applied to the transformed data and whether to use normalized intensities for plotting (parameters n, smooth and normalized in plot_ma(.)).


df %>%
calculate_ma() %>%
plot_ma(n=0.1)


Note that, by default, plot_ma(.) visualizes the plots per sample and we use stat_bin_2d(.) in Figure 2C to illustrate the point density and error distribution across the whole dataset. The MA plot in Figure 2C reveals an erroneous heterozygous cluster where m is close to zero and a is low that we previously showed is due to ADI. ^6^ We subsequently perform sample genotype classification with SureTypeSC using:
clf=system.file('files/rf.clf',package="SureTypeSCR")

df_model = df %>%
       calculate_ma() %>%
       group_by(individual) %>%
       nest() %>%
       mutate(model=map(data, function(df) suretype_model(df,individual, clf)))


The first layer of the classification algorithm (Random Forest) is loaded from the file. Then, the classification model is created per individual sample (group_by(.) and nest(.)) using Gaussian Discriminant Analysis to infer model parameters. ^6^ The Gaussian Discriminant Analysis is conducted per individual sample rather than the combined dataset in order to avoid bias in the scoring function due to potential outliers in the data. The first two parameters of suretype_model(.) are formal and the last parameter defines the classifier (clf) to be used in the first layer (see the reference manual for a detailed description of all available parameters). After unnesting the df_model, the dataframe contains an additional column that contains the SureTypeSC classification score (rfgda_score). We can then apply a threshold (set_threshold(.)) and use MA plot again to observe how SureTypeSC has affected the quality of the data:


df_model_unnested = df_model %>%
unnest(c(data,model))

df_model_unnested %>%
set_threshold(clfcol='rfgda_score',threshold=0.5) %>%
plot_ma()


Figure 2D shows the results from the entire dataset (using stat_bin_2d(.)). Unlike Figure 2C, which contains the data prior to SureTypeSC, the heterozygous cluster (m close to 0 and low a) caused by ADI is effectively removed and the data are concentrated along m = 4 and m = −4 representing homozygous AA and homozygous BB genotypes, respectively.

Finally, we determined the call rate and % of heterozygous SNPs in the data as a function of the used threshold in both SureTypeSC and Illumina's GenCall (rfgda_score and score columns in the data frame, respectively):


callr_calc <- function(.data,algo,threshold)
{
  .data %>%
   set_threshold(clfcol=algo,threshold=threshold) %>%
   group_by(individual,gtype) %>%
   callrate() %>%
   pivot_wider(names_from=gtype, values_from=Callrate) %>%
   mutate(thr=threshold, alg=algo) %>%
   mutate(callrate=AA+BB+AB,thr=threshold,alg=algo)
}

thrs=seq(0.0,0.96,0.01)

suretype=(map(thrs,function(x) callr_calc(df_model_unnested,'rfgda_score',x))) %>% bind_rows()
gencall=(map(thrs,function(x) callr_calc(df_model_unnested,'score',x))) %>%
bind_rows()
performance=bind_rows(suretype,gencall)


Figure 2E confirms that SureTypeSC is more specific towards noise whilst retaining higher call rates as the threshold increases compared to GenCall. This is consistent with our validation study published previously. ^6^

## Conclusions

Although data from single cell genotyping using SNP arrays have been subjected to meta-analysis in the last decade to reconstruct haplotype maps, ^14, 15^ automated analysis has remained challenging. SureTypeSCR is an R package that aims to facilitate single cell SNP array analysis by encapsulating typical parts of the workflow into a common interface by following modern data science principles represented by the tidyverse ecosystem. The algorithm used for genotype classification is state-of-the-art in the single cell SNP array domain and is designed as a plug-in system for the SureTypeSCR package. ^6^ We show typical use on real world data ( Figure 2) with code snippets that demonstrate the functionality of the package. SureTypeSCR offers a single cell genotyping method with good precision in an easy-to-use R package, thus making it suitable for research and potentially clinical applications.

## Data availability

NCBI GEO: Preclinical Validation of a Microarray Method for Full Molecular Karyotyping of Blastomeres in a 24-hour Protocol, Accession number GSE19247: https://www.ncbi.nlm.nih.gov/geo/query/acc.cgi?acc=GSE19247.

## Software availability

• Software and source code available: https://github.com/Meiomap/SureTypeSCR.
• Archived source code at time of publication: https://doi.org/10.5281/zenodo.4963845. ^16^
• License: GNU-GPL-3.
