# Supplementary material for: SureTypeSCR: R package for rapid quality control and genotyping of SNP arrays from single cells
Source: F1000Res. 2021 Sep 21;10:953. [Version 1] doi: 10.12688/f1000research.53287.1 (PMC11401986; doi:10.12688/f1000research.53287.1)
Supplement: Peer review history

# Reviewer report: Reviewer response for version 1

**DOI:** 10.5256/f1000research.56654.r245600

**Published:** 2024-09-14

**Authors:** Kun Lu Lu

**Affiliations:** 1 Southwest University, Chongqing, China

In this study, the authors developed an R package called SureTypeSCR for SNP arrays analysis and single cell genotyping. Although this is an important and original work for single cell genotyping, there are still some suggestions for the manuscript as it stands.

 1.Besides calculating call rate, the number of SNPs and genotyping precision or accuracy per individual also should be calculated.

 2.In the step “data initialization and QC”, the IDAT or GTC files cannot be found in R codes.

 3.In Figure 2A, the genotype reveals a high degree of heterozygosity (AB rates), however, how many heterozygous SNPs were caused by ADI error? In addition, how to detect ADO error due to ADO is common and affects up to 30% of typed SNPs.

 4.In Figure 2E, the authors used different threshold to determine the call rate and percentage of heterozygous SNPs. What does the threshold mean? What is the relationship between the threshold and heterozygous SNPs?

Are the conclusions about the tool and its performance adequately supported by the findings presented in the article?

Yes

Is the rationale for developing the new software tool clearly explained?

Yes

Is the description of the software tool technically sound?

Yes

Are sufficient details of the code, methods and analysis (if applicable) provided to allow replication of the software development and its use by others?

Yes

Is sufficient information provided to allow interpretation of the expected output datasets and any results generated using the tool?

Yes

Reviewer Expertise:

Plant Genomics

I confirm that I have read this submission and believe that I have an appropriate level of expertise to confirm that it is of an acceptable scientific standard, however I have significant reservations, as outlined above.

# Reviewer report: Reviewer response for version 1

**DOI:** 10.5256/f1000research.56654.r245583

**Published:** 2024-03-12

**Authors:** Jason Torres

**Affiliations:** 1 University of Oxford, Oxford, UK

This this article ,the authors describe a newly developed R package - "SureTypeSCR" - that implements a previously-developed machine learning-based procedure ("SureTypeSC") for performing quality control of single-cell genotyping data. This package aims to streamline the quality control procedure (i.e. data formatting, normalisation and visualisation) within a single programming environment. This approach offers the greatest benefit to the analysis of single-cell genotyping data following whole genome amplification (WGA), as WGA introduces genotyping errors that require careful filtering.

 The article provides a clear explanation of the rationale for the SureTypeSCR method, and its application to real-world data of 23 sperm samples from Johnson et al. 2010. My comments are relatively minor, but do require considered responses: 

 1) The authors mention in the introduction that including  "GenomeStudio in a pipeline with large sample batches can be impractical as the data loading process needs to be curated manually."  They further state that they "encapsulated the functionality of SureTypeSC together with automated feature extraction from the raw GTC data into an R package". It is not clear to me if this automated feature extraction addresses the impractical manual curation problem introduced previously. If so, it would be helpful to make the point explicitly. 

 2) In the "Metadata" section, for completeness, it would be helpful to explain the content of the "sample sheet" input file, as all other input file types are address. It may also be helpful to provide example text of the fields present in each data type within Table 1. 

 3) I was not able to successfully install SureTypeSCR within my R environment (4.3.1). During the installation of the python virtual environment, I was notified that the pip install sklearn command failed as sklearn was deprecated and instead required installation of scikit-learn. I then referred to the GitHub page to manually install SureTypeSCR using the command: 

 R CMD INSTALL SureTypeSCR_0.99.0.tar.gz

 However, when I tried to download the tar.gz file from the "current release" page, I encountered "404 - page not found' error. Therefore, I couldn't install and verify the functionality of SureTypeSCR. 

 4) Similar to the other reviewer, it wasn't clear to me why all sperm samples had the exact same "NC" proportion in Figure 2A. Also, it would be informative to know how many SNPs were used in the analyses required to generate the PCA and MA plots. 

 Also, as a minor comment. The last sentence of the abstract states that SureTypeSCR can also be "used for standard genotyping of bulk DNA for batch processing in a single pipeline". However, from what I can discern, this was not explicitly demonstrated in this manuscript. It may therefore be helpful to include additional examples with real-world bulk DNA data. This could be perhaps included as an additional section within the GitHub tutorial. 

 I'd greatly appreciate the authors' responses to these comments. 

Are the conclusions about the tool and its performance adequately supported by the findings presented in the article?

Yes

Is the rationale for developing the new software tool clearly explained?

Yes

Is the description of the software tool technically sound?

Yes

Are sufficient details of the code, methods and analysis (if applicable) provided to allow replication of the software development and its use by others?

Partly

Is sufficient information provided to allow interpretation of the expected output datasets and any results generated using the tool?

Yes

Reviewer Expertise:

Complex trait genetics, genetic epidemiology, molecular epigenomics

I confirm that I have read this submission and believe that I have an appropriate level of expertise to confirm that it is of an acceptable scientific standard, however I have significant reservations, as outlined above.

# Reviewer report: Reviewer response for version 1

**DOI:** 10.5256/f1000research.56654.r94990

**Published:** 2021-10-22

**Authors:** Joris Robert Vermeesch

**Affiliations:** 1 KULeuven, Leuven, Belgium; 2 Department of Human Genetics, Centre for Human Genetics, University Hospitals Leuven, Leuven, Belgium

This paper presents an R package that can contribute to the quality control and genotyping of SNP arrays generated from amplified single-cell DNA. The package builds on a previous R package, SureTypeSC. The package will be useful for laboratories using SNP arrays on amplified single-cell DNA. Overall, the descriptions are very detailed and clear, ready to be used. 

- Figure 2(A) of the paper, why were "NC" values all the same for every sample?
- The software requires extra data files, such as HumanCytoSNP-12v2_H.bpm and HumanCytoSNP-12v2_H.egt. It would be helpful to leave the links for downloading those files.

Are the conclusions about the tool and its performance adequately supported by the findings presented in the article?

Yes

Is the rationale for developing the new software tool clearly explained?

Yes

Is the description of the software tool technically sound?

Yes

Are sufficient details of the code, methods and analysis (if applicable) provided to allow replication of the software development and its use by others?

Yes

Is sufficient information provided to allow interpretation of the expected output datasets and any results generated using the tool?

Yes

Reviewer Expertise:

Genetics, genomics, cytogenetics, embryo, prenatal, structural variation

I confirm that I have read this submission and believe that I have an appropriate level of expertise to confirm that it is of an acceptable scientific standard.
